# Hybrid Polymerisation: An Exploratory Study of the Chemo-Mechanical and Rheological Properties of Hybrid-Modified Bitumen

**DOI:** 10.3390/polym12040945

**Published:** 2020-04-18

**Authors:** Ilya Binti Joohari, Filippo Giustozzi

**Affiliations:** 1 Civil and Infrastructure Engineering, Royal Melbourne Institute of Technology (RMIT) University, 376-392 Swanston St., Melbourne VIC 3000, Australia; 2 School of Environmental Engineering, Universiti Malaysia Perlis, Pusat Pengajian Jejawi 3, Arau 02600, Perlis, Malaysia

**Keywords:** hybrid polymers, rheology, elastomer, plastomer, crumb rubber, bitumen

## Abstract

In this study, the mechanical and rheological properties of hybrid polymer-modified bitumen (PMB) have been investigated. For this purpose, nine different polymers—including crumb rubber, elastomers and plastomers at varying content—were studied to evaluate their mechanical performance as single polymers, first, and as a combination of two or more polymers as a hybrid polymer blend. Subsequently, the hybrid polymer blends were added in a relatively small percentage into the base bitumen to study its influence on the rheological performance of hybrid PMB. The mechanical properties identified from the analysis of the stress-strain curve of the single polymers were the Young’s Modulus, tensile stress, and elongation at break. The chemical structure of the polymer hybrid blends was analysed using FTIR, followed by frequency sweep tests conducted using the dynamic shear rheometer (DSR) to determine the bitumen rheological properties. Results showed that hybrid PMB enhances the viscoelastic behaviour of bitumen at both low and high temperature compared to other PMBs only including single polymers.

## 1. Introduction

Asphalt pavement is highly susceptible to heavy traffic loads and extreme weather conditions. As a material that is classified as viscoelastic (time- and temperature-dependent), asphalt is commonly affected by three main types of distress, which are fatigue cracking, thermal cracking and rutting [1,2]. In recent years, there has been a high demand for modifying bitumen with polymers to improve the asphalt pavement performance at low and high temperatures, simultaneously [3,4,5].

In many cases, the term Polymer-Modified Bitumen (PMB) refers to the mixing of a polymer into the bitumen. The most common practice is by using immiscible polymers to modify the bitumen [6,7,8,9]. Immiscible polymers are polymers that are mixed to obtain a material with combined properties of the polymer and source material. The polymers should be compatible with bitumen and exhibit enhanced performance at both high and low temperatures [10] hence reducing its thermal susceptibility.

Despite numerous polymeric products being produced, only a small number of polymers are being deployed that are suitable to be used as a bitumen modifier [11]. The use of polymers in bitumen usually involves two types, which belong to the plastomer and thermoplastic elastomer families [12,13,14,15]. These two polymer groups enhance the specific properties of the bitumen differently. Plastomer—although many different families of plastomer are available nowadays—typically enhances the stiffness and resistance to permanent deformation against loading, while thermoplastic elastomer provides better fatigue resistance due to its elastic properties [16]. However, the most widely accepted bitumen modifier is the SBS copolymer. 

Styrene-butadiene styrene (SBS) block copolymers are one of the most commonly used thermoplastic elastomers to modify bitumen [17]. The term copolymer is used to describe a polymer chain with two different types of monomers connected. SBS is made up of two monomers which are styrene (hard block) and butadiene (soft block). Both styrene and butadiene have different mechanical behaviours, where the first provides the strength and the latter contributes to the elastic properties [18]. At room temperature, styrene with a glass transition temperature of around 100 °C shows a stiff behaviour, while butadiene, which has a glass transition temperature of around −100 °C, shows a soft and elastic behaviour [19]. The properties of the polymer can potentially be modified by changing the styrene:butadiene ration. A good strength performance displayed by SBS, at both high and low temperature, is necessary to minimize asphalt fatigue and permanent deformation. 

After SBS was developed in 1965, another thermoplastic elastomer—styrene ethylene butylene styrene (SEBS)—was introduced in 1972 [2]. Similar to the SBS, SEBS is also regarded as a styrenic thermoplastic elastomer with an A–B–A block copolymer, where A is polystyrene and B is an elastomer segment (i.e., S–B–S and S–EB–S) [20]. Styrenic thermoplastic elastomers have similar physical properties to rubber although their melt processability is typical of conventional thermoplastics [21]. The presence of the ethylene component in SEBS lowers the crystallization temperature (T_c_), thus changing its behaviour compared to SBS [22]. Over the years, research on modified bitumen with SEBS has shown that it improves resistance at low temperature [23] and reduces susceptibility to thermal degradation [6] and permanent deformation [24].

A plastomer that has gained recognition for application as a modifier in bitumen is polyethylene (PE). PE enhances the rigidity of asphalt, as well as increases the rutting resistance at high temperature [25]. Additionally, PE has the chemical properties of being highly robust and exhibits a longer degradation period compared to other commercial plastics. The most frequently used PEs in PMB are low-density polyethylene (LDPE), linear low-density polyethylene (LLDPE), and high-density polyethylene (HDPE) [26]. LDPE (density of 0.91–0.94 g/cm^3^) consists of carbon atoms attached to long chains of PE. On the contrary, HDPE (density of 0.94 g/cm^3^) has fewer and short-chain branching [27]. A slightly different version of LDPE is L-LDPE, which has a density similar to LDPE (0.918 g/cm^3^), but its molecular chain linearity is similar to HDPE which is shorter branching compared to LDPE [28]. In polymers, branching plays an important role as a structural parameter; long-chain branching exhibits better-melting properties and processability, while short-chain enhances mechanical and thermal properties [29].

To reduce the environmental impacts of road construction, bitumen modification with recycled linear low-density polyethylene (RLLDPE) has been developed to promote recycling and reusing plastic waste. The properties of recycled polyethylene highly depend on the processing conditions; whereby high processing temperature and residence time will reduce mechanical properties, especially elongation at break, as well as enhancing degradation of the material [30]. It is also to be noted that recycled polyethylene contains surface contaminants and other components scattered in the polymeric matrix, as well as those derived from degradation such as oxidative degradation due to their exposure to weathering [31].

Ethylene-vinyl acetate (EVA) copolymer is another commonly used plastomer in the PMB industry; it is composed of two segments, ethylene, and vinyl acetate. Both segments complement each other where the ethylene segment is nonpolar-but-crystalline, compared to the polar-but-noncrystalline vinyl-acetate [32]. EVA exhibits lower crystallinity compared to LDPE due to the presence of acetate groups [33]. Nevertheless, EVA—being a semi-crystalline copolymer—enhances the physical, chemical and morphological properties of PMB by forming a rigid, tough and three-dimensional network to resist distresses in asphalt binder [32,34]. 

In addition to the plastomer and elastomer groups, several polymers are now being produced from recycled materials. Nowadays, the use of recycled material in asphalt applications is gaining continuous attention [35]. Historically, crumb rubber (CR) is one of them, which is produced from recycled and processed automotive tires. CR has been proven to improve bitumen resistance against fatigue, rutting and thermal cracking at both high and low temperatures [36]. Through the wet process, CR is blended into the hot bitumen; the rubber then swells due to the absorption of aromatic oils from the bitumen. As a result of the depletion of aromatic oils in the bitumen, the modified bitumen becomes stiffer [15]. Even though CR is recognized as a low-cost (i.e., compared to the costs of other non-recycled polymers) and environmentally friendly option for modifying bitumen [37], CR displays low resistance to heat, which is a disadvantage as it minimizes the pavement service-life period and, potentially, the end-of-life recyclability [38]. Therefore, the combination of CR and various types of polymers has been studied and has shown significant improvements in the bitumen properties [39,40,41]. 

This study aims to investigate the effects of different hybrid blends of polymers and CR at different percentage combinations on the mechanical and rheological properties of the immiscible polymers alone. For this purpose, nine different polymer blends were selected, with three different percentage combinations for each blend. These blends were tested for tensile strength tests to study the mechanical properties of the polymers. The optimum result from each blend was later tested using Fourier-transform infrared spectroscopy (FTIR) to study their chemical properties, followed by their addition to base bitumen for PMB rheological investigation.

## 2. Materials and Methods 

### 2.1. Materials

To study the effect of different hybrid blends of polymers, seven different polymers were combined at different percentages. These combined copolymer blends included SBS, SEBS, EVA, LDPE, LLDPE, RLLDPE and CR, as shown in Table 1, whereby “B” refers to blend and “M” to mix (e.g., B1M1 is blend 1 mix 1). The content of each polymer for the hybrid blends was selected based on commonly used polymer contents to modify bitumen by previous research [42,43,44,45,46]. A recent review by Behnood and Gharehveran stated that the most commonly used polymer additives are between 4–6% by weight of asphalt binder. Higher content of polymer additives are considered to be less economical and might pose other problems such as phase separation and loss of workability [1]. However, in case of CR, Xu et al. stated the addition of approximately 15–20% CR into the bitumen is considered optimal depending on the source of base bitumen and performance grade [47]. The properties of each polymer are shown in Table 2.

### 2.2. Methods

**Figure 1:**
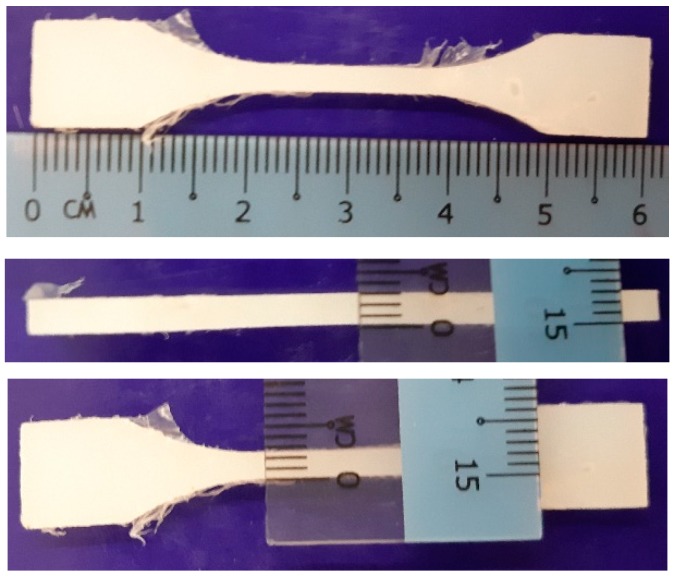
Dimensions of the dog-bone tensile samples.

The polymer samples were prepared using the Haake Polylab Rheomix (Thermo Fisher Scientific, Waltham, MA, USA) at 170 °C and 50 rpm. The following procedure was adopted. First, two polymers at different content were weighed to a total of 50 g. Both polymers were blended using the Rheomix for 15 min to ensure optimum homogeneity between the polymers. This step was followed by compressing the blended polymers into a 2.5-mm-wide and 3-mm-thick tensile dog-bone mould (as shown in Figure 1) at a temperature of 170 °C, with a force of 90 kN. The samples were given a stress-relaxation time of at least 24 h before any test was being conducted. 

The tensile strength test was conducted in accordance with ASTM D638 using the Instron 5900 with a loading rate of 50 mm/min. The load cell capacity was 2 kN. Five specimens were prepared for every polymer blend, as required by the standard. Single polymer specimens were also tested as control samples. The optimum tensile strength performance was evaluated as a selection parameter for every hybrid polymer combination, which was then selected to undergo further FTIR test. 

Out of 27 blends conducted for the tensile strength test, nine optimum blends were selected based on mechanical strength and elongation potential to be tested for FTIR. FTIR was conducted to further understand the chemical compositions of the hybrid polymer blends. This instrument is widely used to represent the composition, formation, crystallinity, and behaviour of polymeric materials [42,48]. The chemical structure of the polymer blends was analysed using the Perkin Elmer Spectrum 100 (FT-IR Spectrometer, PerkinElemer, Waltham, MA, USA). The wavenumber ranges from 4000 to 600 cm^−1^ with the number of scans of 32 times. The resolution adopted was 4 cm^−1^. 

Based on the nine optimum polymer blends, hybrid PMBs were prepared using Silverson Shear Mixer (Silverson, USA) equipped with a slotted disintegrating head. The base bitumen was firstly heated to 180 °C prior to the hybrid polymer addition. The mixing process was conducted for one hour at 3500 rpm to create a homogenous blend between the bitumen and polymers. The hybrid blends were then stored at 160 °C in the oven for another hour to cure before any experimental test was conducted.

**Figure 2:**
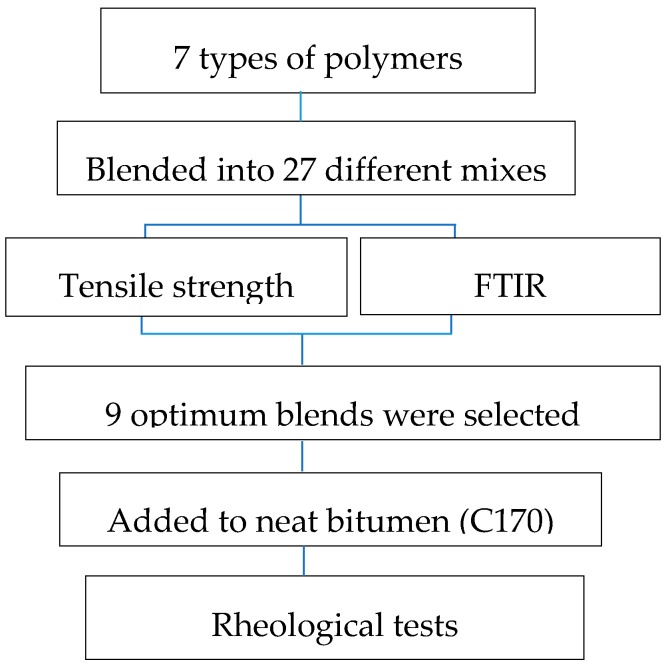
Research method summary.

Frequency sweep tests were conducted to evaluate the linear viscoelastic behaviour of the modified bitumen with the optimum polymeric blends using the dynamic shear rheometer (DSR). The base bitumen C170 was used for all nine PMBs. Oscillating shear stresses were applied at 0.1% controlled strain, while the frequency varied from 0.1 Hz to 15 Hz. The test was conducted at 5 °C, 15 °C, 30 °C, 50 °C, and 70 °C. Modified bitumen samples of 8 mm diameter with 2 mm thickness were used for lower temperatures (5 °C and 15 °C), while 25 mm diameter and 1 mm thickness were used for higher temperatures (30 °C, 50 °C, and 70 °C). The phase angle, complex modulus, and master curve of hybrid-modified bitumen were developed based on the frequency sweep results. The overall polymer loading in hybrid-modified bitumen was less than 6%; this value is in agreement with many types of polymer-modified bitumen (i.e., using a single type of polymer) currently available on the market. It was the purpose of this experimental campaign to verify how a partial substitution of standard polymers (i.e., linear SBS) with plastomers (virgin or from recycled plastic waste) and/or crumb rubber would benefit the overall bitumen performance. It is thus anticipated that no modification of the current testing standards is needed to evaluate the performance of the combination of two or more polymers with bitumen. The total polymer loading is also expected not to significantly modify common practices to lay asphalt mixes including hybrid-modified bitumen although further study is needed for hybrid-asphalt mixes. Figure 2 outlines a summary of the laboratory tests conducted in this study. 

## 3. Results and Discussion

### 3.1. Mechanical Analysis of Hybrid Polymers

**Figure 3:**
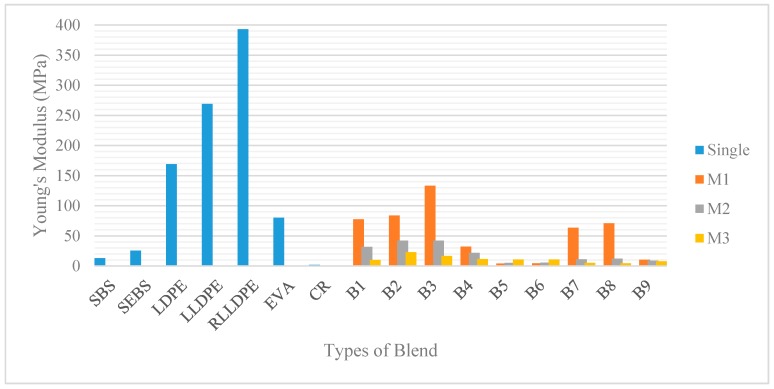
Young’s Modulus of single and hybrid polymers.

**Figure 4:**
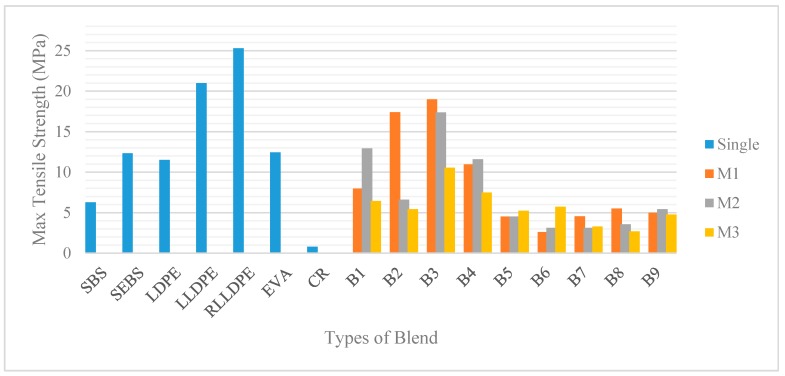
Maximum tensile strength of single and hybrid polymers.

**Figure 5:**
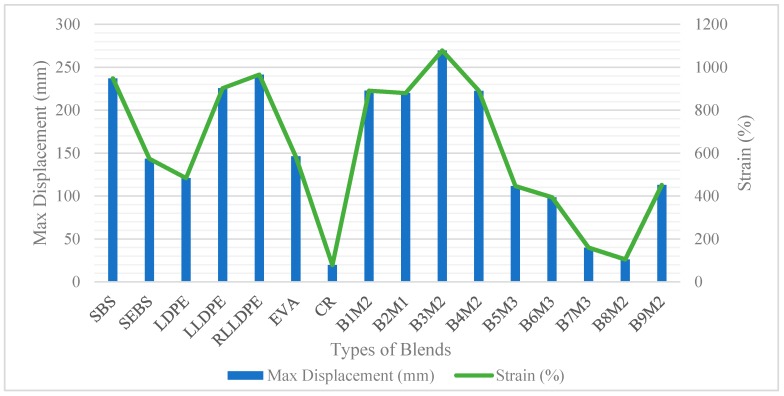
Elongation and strain of single and optimum hybrid polymers.

The mechanical properties of the single polymers and their polymeric blends were measured based on their tensile strength at 25 °C (ASTM D638). From the stress-strain curve, the tensile mechanical properties identified were the yield point, Young’s Modulus, stress and elongation at break. The results obtained are summarized in Figure 3, Figure 4 and Figure 5.

#### 3.1.1. Tensile Test: The Interaction between Plastomers and SBS

Plastomers (i.e., LDPE, LLDPE, EVA) have been suggested to increase the stiffness of the material [43], while elastomers (i.e., SBS, SEBS) are known to contribute more to the elastic behaviour [44]. Young’s modulus is used to characterize the stiffness, while the elongation reflects the elastic characteristics. Based on the Young’s modulus values displayed in Figure 3, plastomers—specifically LDPE, LLDPE, and RLLDPE—showed a very high stiffness value compared to the elastomers, with 169, 269, and 393 MPa, respectively. LDPE possesses long chain branching and is formed by a high-pressure polymerization process. This process caused the incapability of the molecules to pack closely together during crystallization. Consequently, this results in low crystallinity, which contributes to the low tensile strength [45]. The properties of the LLDPE and RLLDPE, which are synthesized from the Ziegler-Natta process by incorporating α-olefins, are also highly dependent on the molecular branching. In contrast, the short-chain branching is molecularly heterogeneous and results in a complex crystallinity, with improved tensile strength [46]. 

Generally, crystalline polymers have high tensile strength, tensile modulus, and rigidity, whereas SBS contributes to the elasticity and toughness [49]. B1, B2, B3, and B4 are the polymeric blends between plastomers and SBS with three different percentage combinations (i.e., M1, M2, and M3). M1 with 3% plastomer content yields the highest value for Young’s modulus. Subsequently, Young’s modulus was noted to be decreasing when reducing the plastomer content and raising the SBS content. In comparison with the polyethylene, EVA was observed to display the lowest stiffness due to the presence of the acetate groups which lowers its degree of crystallinity [33]. All in all, the four blended plastomers with SBS failed to compete with stiffness values of the singular plastomers only. This is obviously due to the low stiffness value of SBS, which was recorded to be only 13 MPa. SBS displays a two-phase morphology made of two glassy polystyrenes (PS) segments which are connected by a rubbery polybutadiene (PB) segments [50]. This gives dominant toughness and elastic characteristics which can be seen from the maximum tensile strain and elongation. From Figure 5, SBS, LLDPE, and RLLDPE displayed comparable displacement values of 237, 225, and 241 mm, respectively. B3M2 which consists of 3.5% SBS and 2% RLLDPE resulted in the greatest displacement value (270 mm) compared to the rest of the polymeric blends and their respective singular polymers. The presence of carbon black, which also acts as a filler [51] in the RLLDPE, seemed to improve the overall mechanical performance of the blend in contrast with the LLDPE blend.

Although the displacement value of the SBS and polyethylene are quite equivalent, the mode of break between the two is distinguishable. Being an elastomer, SBS elongated and returned to its original shape, whereas the plastomer failed to do so as the semi-crystalline molecules slid and became entangled, thus breaking apart when the tensile strength limit was reached. The breaking mode of each polymeric blends highly depends on which polymer content is more dominant between the two. For further investigation, B1M2, B2M1, B3M2, and B4M2 were selected as the optimum content based on their overall mechanical performances. 

#### 3.1.2. Tensile Test: The Interaction between Thermoplastic Elastomers and CR

CR is known as a material that absorbs liquid and swells, depending on the temperature, nature, and viscosity of the liquid/solvent [52], which makes it a suitable recycled material for enhancing bitumen performance. However, on its own, CR lacks mechanical performance, as seen in Table 3. SBS and SEBS are both regarded as styrenic thermoplastic elastomers with an A–B–A block copolymer, where A is polystyrene and B is an elastomer segment (i.e., S–B–S and S–EB–S) [20]. Styrenic thermoplastic elastomer has similar physical properties to rubber, but melt processability typical of conventional thermoplastics [21]. In 2011, Kök and Çolak did a study on CR and SBS modified bitumen separately. It was concluded that the CR content of CR-PMB must be higher in order to achieve equivalent performance as SBS-PMB since CR and SBS exhibit different properties despite both being categorized as elastomers [53]. Hence, the selected contents of SBS and SEBS were both 2%, 3.5%, and 5%, while those of CR were 5%, 12.5%, and 20%. 

From Table 3, it can be found that increasing contents of both SBS and SEBS improved the mechanical properties when blended with CR. Compared to SBS, the presence of the ethylene component in SEBS lowers the crystallization temperature (T_c_), thus differentiating the two [23]. This reflects the higher stability of the SEBS molecules when compared to SBS. In the case of Young’s modulus, the maximum content of 5% SBS and SEBS added to the 5% CR (B5M3 and B6M3) displayed comparable increase factors of 6.12 and 6.20, respectively. However, the ethylene component of SEBS proved to slightly increase B6M3 with an increase factor of 7.25 compared to B5M3 (5.72). This trend was also observed for the maximum displacement, whereby the SEBS-CR blend (B6M3) had a higher increase factor compared to the SBS-CR blend (B5M3), with recorded values of 3.18 and 5.03, respectively. The increment of both maximum tensile strength and displacement indicates that SEBS has a superior elastic behaviour compared to SBS. Aside from that, the overall mechanical performance is considered to be low mainly due to the CR content. This is explained by the rubber phase in CR, which is highly cross-linked, limiting the rubber molecules’ freedom to entangle with other polymer molecules. Consequently, this results in low interfacial adhesion between the two, which is observed through the tensile elongation at break [54]. A similar phenomenon is observed when CR is combined with plastomers. All in all, B5M3 and B6M3 were chosen as the optimal blends for further testing.

#### 3.1.3. Tensile Test: The Interaction between Plastomers and CR

Plastomers such as polyethylene (PE) are said to provide rigidity to the binder and minimize deformation under load, due to the sliding of the polymeric chains and entanglement occurring within the material [55]. Therefore, plastomers were added to CR with the aim of improving mechanical properties such as stiffness when using recyclable material to modify bitumen. The selected plastomer contents of 1%, 2%, and 3% were based on research conducted by González et al. (2004), where they concluded that modifying bitumen with 1% was more storage-stable compared to 3%, which displayed phase separation [51]. 

Table 4 shows the effect of combining plastomers (i.e., LLDPE and RLLDPE) with CR in terms of mechanical performance. In general, both plastomers play their role by significantly increasing the Young’s modulus value with increasing content of M3 (1%), M2 (2%), and M1 (3%), respectively. On the contrary, the increasing content of plastomer blended with CR showed a decreasing trend for the maximum tensile strength and displacement recorded. This shows that plastomers predominantly contributed to the stiffness and rigidity but played an insignificant role in improving the elastic behaviour of the blended polymers. It is noteworthy to point out that at 3% LLDPE and RLLDPE, the maximum displacement recorded was an increase factor of less than 1. This shows that at 3%, both blends were too stiff, reducing the minimum elasticity which is contributed by the 5% CR content. The results obtained were in line with a study done by Yan et al. (2015), where they concluded that the mechanical improvement was highly influenced by the polymer content [56]. From the analysis of these two interactions, B7M3 and B8M2 were chosen as the optimum blends for further testing. 

#### 3.1.4. Tensile Test: The Interaction between Thermoplastic Elastomer, Plastomer, and CR

Through microstructure and thermal analysis, Formela et al. (2016) claimed that the physical interaction between the polymer additives and bitumen are limited with increasing CR content. The cross-linked structure and low flowability of the CR were said to cause this phenomenon [57]. In another study, Xia et al. (2018) investigated the effects of using both SBS and PE in bitumen modification, mainly focusing on viscoelastic phase separation. It was found that both blends displayed viscoelastic phase separation, whereby the network structure was created by the PE-rich phase and SBS-rich phase, due to the immiscibility between the two blended polymers [58]. These two studies are among many that have suggested the use of thermoplastic elastomer, plastomer, and CR in one blend to minimize such drawbacks. Therefore, in this study, B9 was prepared by mixing 5% SBS and 5% CR with three different EVA contents, namely M1 (3%), M2 (2%), and M3 (1%). The increment of EVA content was observed to increase Young’s modulus values, which are 7.87 MPa, 8.87 MPa, and 10.21 MPa, respectively. However, B9M2 was found to provide the highest tensile strength of 5.42 MPa. All three blends within B9 show improved tensile strength compared to plastomer–CR blends, but lower than the plastomer–SBS blends. This also reflects the lower displacement measured by the three B9 mixes compared to the plastomer–SBS blends, which indicates that the elastic behaviour has been reduced. Nonetheless, the mechanical performances are considered satisfactory and B9M2 was selected to be the optimum content between the three. 

### 3.2. Chemical Analysis of Hybrid Polymers

FTIR is used to determine the structural characteristics of the hybrid compounds as all functional groups vibrate at a specific wavenumber [59]. Specifically, the FTIR spectra provide information about the composition and structure of a particular compound due to the differences in absorption of energy by the molecules and their infrared radiation, which can be used to predict chemical and physical properties of the compound.

**Figure 6:**
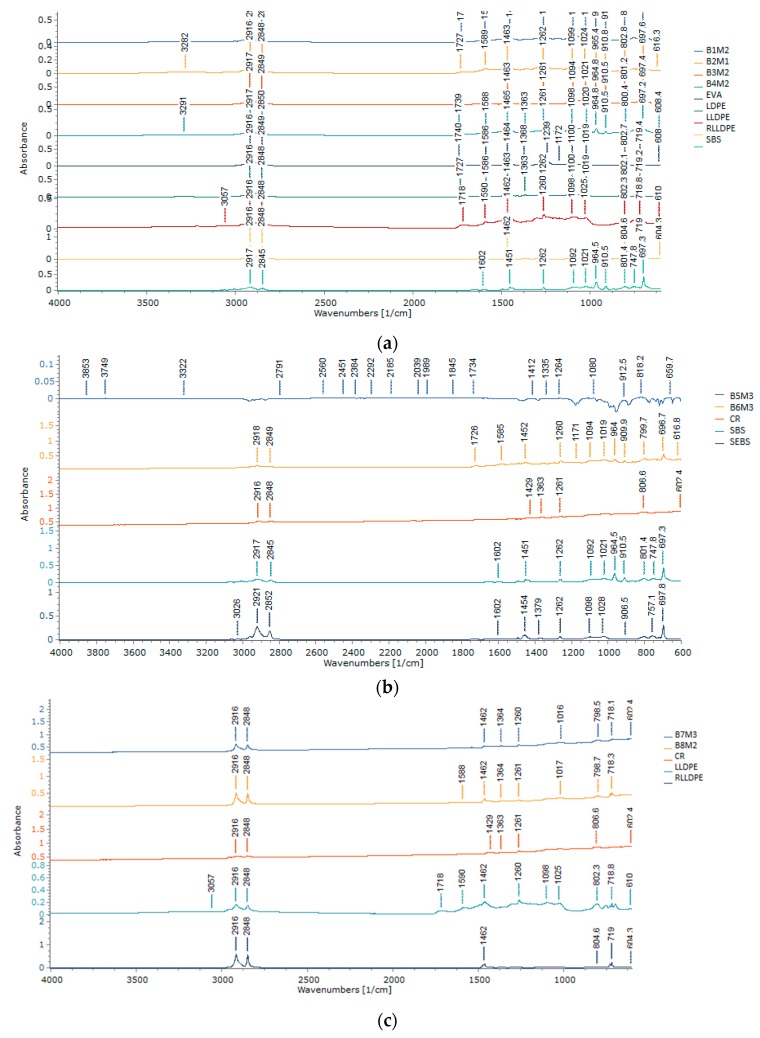

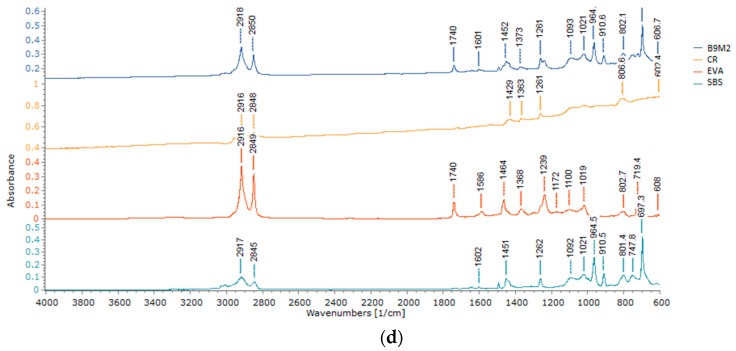
(**a**) FTIR spectra of plastomer–SBS hybrid blends; (**b**) FTIR spectra of elastomer–CR hybrid blends; (**c**) FTIR spectra of plastomer–CR hybrid blends; (**d**) FTIR spectra of the SBS–CR–EVA hybrid blend.

Figure 6a–d illustrates the FTIR spectra obtained for the nine optimum blends selected based on the mechanical properties obtained in the previous section and already discussed. It was observed that the crumb rubber and all polymeric materials exhibit two strong intensity bands between wavenumber 2800 to 3000 cm^−1^. According to Gulmine et al. [42], the bands at 2919 and 2851 cm^−1^ indicate CH_2_ asymmetric and symmetric stretching, respectively. Because all the tested single polymers exhibit these two wavebands, the hybrid blends combing the two materials also showed a similar band.

Bending deformation of bands 1473 to 1463 cm^−1^ was observed in singular plastomers specimen (i.e., EVA, LDPE, LLDPE, and R-LLDPE), as well as all blends containing these plastomers. In polyethylene, bands 2927 cm^−1^ and 724 cm^−1^ correspond to C–H strength and the rocking mode of the CH_2_ group, respectively. All the blends containing two polymeric materials showed that the blend shares the chemical composition of the two original materials, but with lower band intensity. However, Blend 4 with EVA and SBS shows the presence of a new peak at 3291 cm^–1^. The band between 3200 and 3500 cm^−1^ corresponds to either the O–H or N–H functional groups. 

Research by Appiah et al. [60] found that the characteristic frequency of band 1465 to 1375 cm^−1^ corresponds to CH_2_ and CH_3_. This was observed in all the single polymers, as well as their hybrid blends. Band 650 to 1000 cm^−1^, in particular, is used to differentiate LDPE from LLDPE. LLDPE should display a similar and weaker intensity at band 890 (vinylidene group) and 910 (terminal vinyl group) cm^−1^, compared to LDPE, where the 890 cm^−1^ should be predominant [61]. This can be observed in Figure 6a,c.

A strong peak at 964 cm^−1^ in SBS-only specimens was in line with the results obtained by Zhang and Chu [62], where it was mentioned that this peak corresponds to the bending vibration of C–H butadiene double bonds of the SBS molecule chain (–CH=CH–). This is also in agreement with Dong et al. [63], who observed a band of 699 cm^−1^, further labelled as the band for the styrene segment.

### 3.3. Rheological Analysis of Hybrid-Modified Bitumen Blends

Although this study focused on the chemo-mechanical behaviour of single and hybrid polymers, the rheological phase of the experimental plan was essential to evaluate the potential response of hybrid polymers in the road environment. Specifically, the nine optimum blends were mixed with neat C170 bitumen. ‘C170′ is the denomination used by the Australian standards to identify a type of bitumen with a viscosity of 170 Pa·s at 60 °C; this typically corresponds to a penetration grade of 70/100. The softening point for the base C170 bitumen was 47 °C, while the penetration value was 71. 

In characterizing the linear viscoelastic behaviour of the material, both low- and high-frequency tests were conducted. Low frequencies reflect the behaviour of the pavement mixture at slow changes of stress, which occur for slow traffic. On the other hand, high frequencies reflect the behaviour of the pavement mixture for fast traffic [47].

#### 3.3.1. Phase Angle

**Figure 7:**
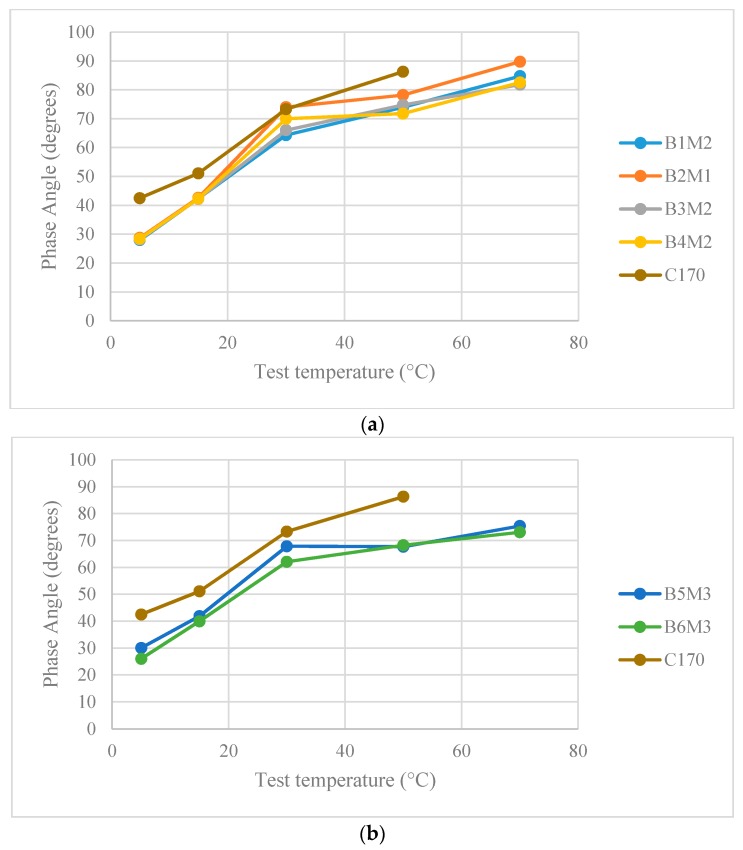

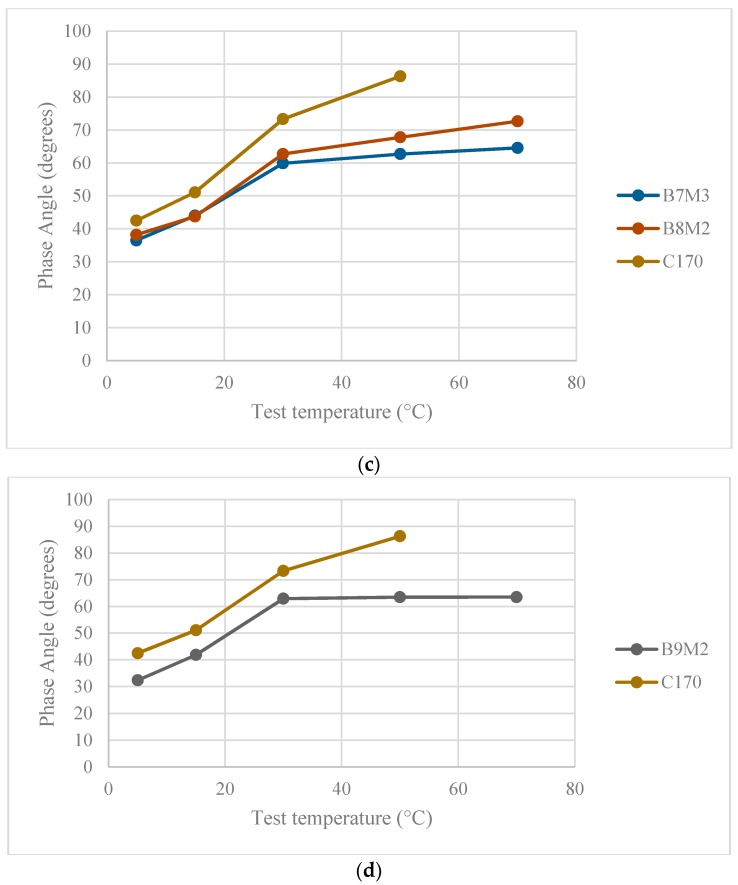
(**a**) Phase angle of plastomer–SBS hybrid blends; (**b**) Phase angle of elastomer–CR hybrid blends; (**c**) Phase angle of plastomer–CR hybrid blends; (**d**) Phase angle of SBS–CR–EVA hybrid blend.

The phase angle is analysed to study the relationship between viscoelastic properties and temperature of the modified bitumen. A perfectly elastic material has a phase angle of zero, while the phase angle approaching 90° indicates a viscous material. The phase angle of a typical asphalt binder ranges between 0 to 90° at in-service temperature conditions [47]. The test results in Figure 7 show the phase angles of the optimum hybrid-modified bitumen blends from 5 °C to 70 °C, at a frequency of 10 Hz. Compared to the neat bitumen, all hybrid PMB blends reduced the phase angle readings at high temperature. This indicates that all optimum polymeric blends enhanced the elastic behaviour of the base bitumen. In addition, an increase in temperature above 30 °C does not correspond to a rapid increase of the phase angle hence confirming the reduced thermal susceptibility commonly ascribed to polymer-modified bitumen. It is noteworthy to mention that the influence of polymer on the rheological behaviour is more predominant at higher temperature due to the low viscosity of the base bitumen; this allows the influence of the elastic network of the polymers to dominantly modify the binder [64].

B2M1, where the bitumen was modified with 2% SBS and 3% LDPE, enhances the viscoelastic properties of the base bitumen but with minimal changes compared to other polymeric blends. For plastomer–SBS blends (B1, B2, B3, and B4), B1M2—a combination of SBS and LDPE—had the highest phase angle at 70 °C, followed by B4M2 (SBS and EVA) and B3M2 (SBS and LLDPE), respectively. This trend verifies that M2 with 2% plastomer and 3.5% SBS is the most compatible in improving the viscoelastic behaviour, compared to B2M1, which contains 3% plastomer content. The higher plastomer content could possibly increase the stiffness although it was found to reduce the elastic response of the particular sample. 

The results for the elastomer–CR blends (B5 and B6) at 70 °C are comparable due to phase angle values of 75° and 73° for SBS and SEBS blends, respectively. At lower temperatures (i.e., 5 °C), B6M3 with 5% SEBS and 5% CR had the lowest phase angle values among all nine optimum hybrid PMB. This indicates that this polymeric blend shows significant elastic behaviour at low temperature, although stiffness and creep relaxation should be also controlled in order to avoid brittleness. For plastomer–CR blends (B7 and B8), B8M2 with the RLLDPE-CR combination behaves in a more viscous manner at 70 °C compared to B7M3 (LLDPE-CR combination). However, the phase angle measured at low temperature was generally similar for both. This correlates well with the single polymers tests where the recycled form of LLDPE showed greater stiffness, and improved elongation and tensile strength.

The hybrid blend (B9M2) containing SBS, EVA, and CR resulted in the lowest phase angle at 70 °C (less thermo-susceptible) among the nine blends. This indicates that this hybrid blend is the most elastic at high temperature, which is very beneficial for improving the overall pavement performance and recovering quickly from deformation at high temperature. At low temperature, B9M2 lies in the intermediate range when compared to all nine hybrid blends. Nonetheless, the phase angle of the three-component blend is higher than all plastomer–SBS blends, which shows that the elastic response increased when CR is added. 

#### 3.3.2. Master Curve of Complex Modulus

**Figure 8:**
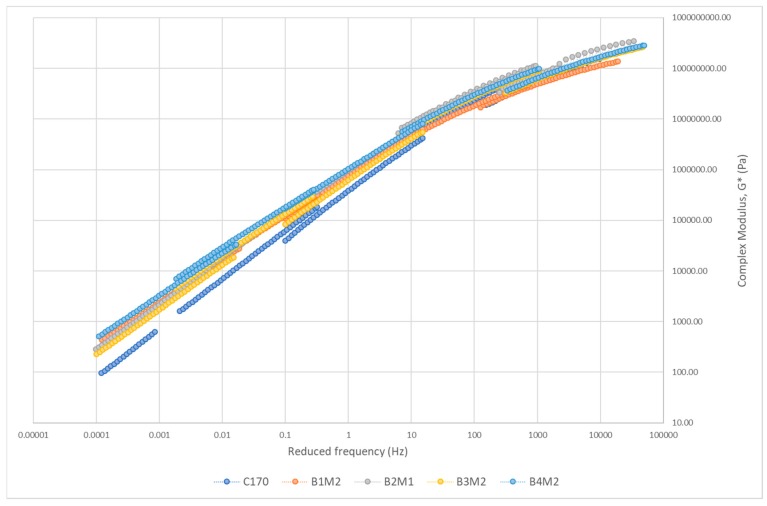
Master curve of plastomer–SBS hybrid blends.

**Figure 9:**
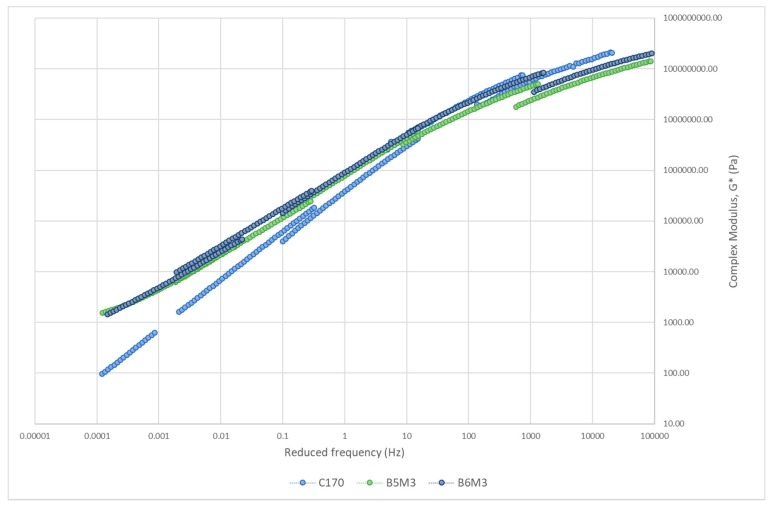
Master curve of elastomer–CR hybrid blends.

**Figure 10:**
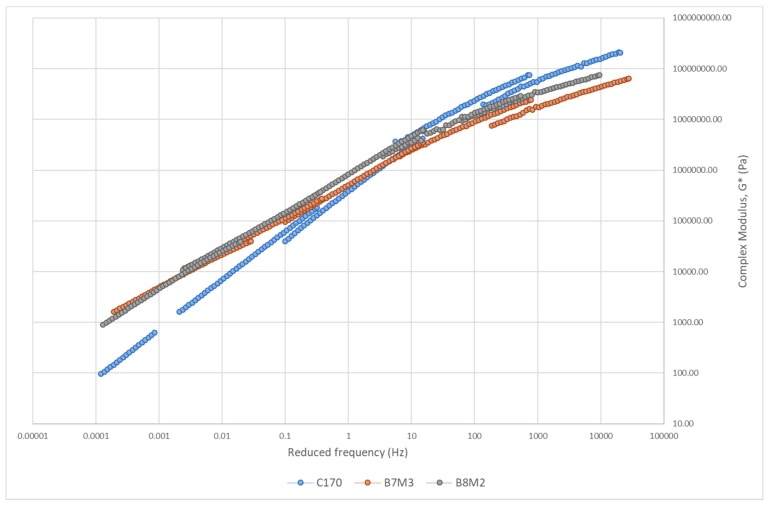
Master curve of plastomer–CR hybrid blends.

**Figure 11:**
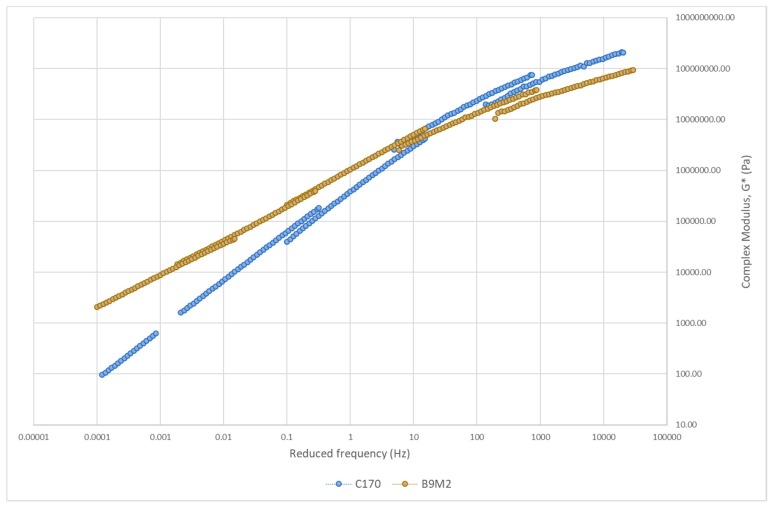
Master curve of the SBS–CR–EVA hybrid blend.

The master curve of asphalt binders is constructed to study the relationship between binder stiffness and reduced frequency over a range of temperatures and frequencies [65]. Figure 8, Figure 9, Figure 10 and Figure 11 show the master curve of clustered optimum hybrid PMB blends using the Christensen-Anderson Model [65]. In short, this method allows the construction of a single master curve through the combination of several measured complex modulus (G*) for specific temperatures, by shifting the isothermal curves to a reference temperature (i.e., 30 °C). In general, all master curves displayed sigmoidal trends with comparable behaviour to the neat bitumen at low temperature (high frequency). This assimilates the stiffness values at the glassy temperature of the bituminous material, whereby it is commonly indicated by a horizontal asymptote ranging between 1 and 1.5 GPa [66]. In contrast, all hybrid PMBs behave differently at high temperatures (low frequencies) compared to the neat bitumen. Accordingly, the rheological behaviour of the neat bitumen is significantly affected when modified with various types of polymers. 

The master curve for plastomer–SBS hybrid blends at various loading frequency is shown in Figure 8. It can be seen that the value of G* at low temperature (high frequencies) is quite similar to the neat bitumen, or slightly reduced. In the high-temperature domain (low frequencies), however, all plastomer–SBS hybrid blends displayed an increased G* value, which highlights the stiffer behaviour of the individual plastomers. These results are in agreement with Young’s modulus values (Figure 3), whereby B1, B2, and B3 show the greatest stiffness. Although B4—blended SBS–EVA—displayed the lowest Young’s modulus value among all plastomers, the master curve showed that its stiffness behaviour at high temperatures is equivalent to the polyethylenes. Such behaviour is due to the fact EVA contains acetate groups which lower its crystallinity. This means that the semi-crystalline structure of the EVA completely melts at a much lower temperature [64] compared to the polyethylenes, hence resulting in better dispersion within the neat bitumen. Essentially, a higher stiffness value than neat bitumen is required at high temperatures because it reflects the PMB potential to minimize susceptibility to permanent deformation (rutting). A study by Xia et al. (2018) claimed that PE content is capable of absorbing the heat developed by the temperature raised close to its melting point, producing a plateau for G* [58]. It can be concluded that the plastomers displayed valuable high-temperature performance but minimal improvement in the elastic properties at low temperature. 

On the other hand, Figure 9 shows the master curve for elastomer–CR hybrid blends. Small differences can be seen at low temperature (high frequencies), although both elastomer–CR hybrid blends slightly decreased the stiffness value. This result also points out the good agreement between the experimental tensile strength tests previously conducted. With reference to Table 3, combining both SBS and SEBS with CR proved to increase the maximum tensile strength and elongation, at increasing elastomer content. The dominant butadiene and ethylene-butadiene content, which contributes to the high elastic response, reduced the stiffness and increased flexibility required to overcome thermal cracking (low temperature) and fatigue cracking (intermediate temperature) [67]. At high temperature (low frequencies), both elastomer–CR hybrid blends interestingly increased their G* value more than all the plastomer–SBS hybrid blends, with measured G* values having more than a 1 kPa increment. This is due to the compatibility and interaction between the elastomer, CR, and bitumen, where the elastomer and CR are swollen by saturates and aromatics from the bitumen allowing these amorphous polymers to blend homogeneously. The miscibility between the elastomers resulted in better performance, including a much more horizontal asymptote at the intermediate temperature range compared to the neat bitumen. This result is similar to the results recently obtained by Lin et al. [68]. Despite improving both low- and high-temperature performance, elastomers were claimed to create a phase separation during storage period due to the elastomer-phase having lesser density than the bitumen-phase [69].

The master curve of plastomer–CR hybrid blends plotted in Figure 10 also shows a distinguishable trend compared to the neat bitumen. Both B7M3 and B3M2—LLDPE–CR and RLLDPE–CR combinations, respectively—showed increased stiffness at low frequencies and decreased stiffness at high frequencies. As previously discussed, it is evident that the two plastomers enhance the performance at high temperature, whereas the CR contributes to the elasticity at low temperature. The master curve’s trend at lower frequency is relatively similar for both hybrid blends, followed by the intermediate temperature range also displaying a much more horizontal asymptote, and the high-frequency G* value being lower than that for neat bitumen. The described trend indicates that plastomer–CR hybrid blends also improved the low- and high-temperature performance of the neat bitumen. It was stated that the crystalline segments of both plastomers should be considered as high-strength filler in the bituminous mixture, promoting the improvement of mechanical properties at most service temperatures [70]. This verifies the results obtained from the tensile strength test for the hybrid blends, in which the singular LLDPE and RLLDPE showed the greatest stiffness and maximum tensile strength. With respect to polyethylenes, it was also mentioned that the linear viscoelastic properties of the bitumen are influenced by the presence of PE-phase. Depending on the polyethylene content, this phase can potentially become a continuous phase and is able to swell with the maltenes in the bitumen. However, the polymer structure of a PE-rich phase collapse when reaching its melting temperature, subsequently shifting the melting temperature of the polymer phase, approaching lower values [70]. This can be translated into a higher phase angle (approaching 90°) and more viscous behaviour at high temperature. To conclude, the content of the polyethylene highly influences the viscoelastic behaviour of the modified bitumen.

As plastomers predominantly contribute to increased rutting resistance, their elastic response at lower temperatures is still limited. Meanwhile, elastomers promote good elasticity and stiffness but are easily susceptible to storage stability issues. Difficulty in storage is also a problem for CR-modified bitumen due to the cross-linked structure of the CR [57]. Therefore, B9M2, which is a blend of SBS, EVA, and CR, is expected to potentially overcome the limitations of hybrid PMB with only two types of polymer. Compared to all nine hybrid PMBs, Figure 11 clearly shows that this hybrid PMB with three different types of polymer enhances the low- and high-temperature performance compared to the other blends. It can be seen that the master curve is more plateaued compared to the neat bitumen. A plateaued or horizontal asymptote master curve is appreciated for predicting the viscoelastic behaviour of pavement at various temperatures and loadings. The content of B9M2 is made up of more elastomers (5% SBS and 5% CR) compared to EVA (2%). However, the 2% plastomer content was seen to shift the master curve upwards at high temperature (low frequencies) more than the downward shifting at low temperature (high frequencies) due to both elastomers. It should be noted that the key parameter in using EVA relies on the total crystalline fraction [71], and will differ depending on the vinyl acetate (VA) content [32]. All in all, B9M2 had the best performance at low, intermediate, and high temperature, as demonstrated by the rheological assessment.

### 3.4. Implementations and Challenges of Hybrid PMB

Based on the laboratory tests conducted in this study, it can be concluded that the hybrid polymer blend is an effective modifier for enhancing bitumen performance. Despite the outcome of providing lower susceptibility to temperature variations, as well as improving resistance towards deformations at higher temperatures, implementing hybrid PMB for pavement applications can still be a challenge. For mass production of hybrid PMB, economical aspects and workability requirements including the feasibility of producing, handling, pumping and compacting [72] must be taken into consideration. Nevertheless, the use of hybrid bitumen blends has the potential to calibrate the use of PMB for the specific application by including a set of polymers that can tackle certain climate conditions, withstand heavier than usual traffic, or a combination of both.

In addition, implementing hybrid PMB also requires the study of bitumen ageing, which can occur during mixing, construction process and long-term service of the pavement. The factors influencing ageing can include the content and characteristic of base bitumen, production-related factors (i.e., temperature and time) [73], as well as the characteristics of the polymer blends, hence making the ageing process very complex. While this study did not focus on the ageing aspect of bitumen, the factors contributing to the ageing of the polymeric system have been previously reported by some researchers [73,74,75,76]. A study using a combination of SBS and EVA indicated that PMB resists the formation of carbonyl and sulphoxide compounds, which improves ageing resistance [75]. Another study suggested the usage of antioxidant in polymer-modified bitumen to minimize ageing as it can hinder the formation of peroxides or by absorbing free radicals [74].

Finally, storage stability would deserve more attention due to the possible segregation issues between the bitumen and the polymers blend [77]. The use of compatibilizers could also be object of future studies.

## 4. Conclusions

This paper evaluated the effects of combining different polymers and their hybrid blends to modify bitumen and the potential of developing hybrid PMBs that combine the characteristics of different polymer groups. Based on the results of this study, the following findings and conclusions can be drawn.

- Based on the tensile strength, the plastomer–SBS hybrid blends have the greatest capability in increasing the Young’s Modulus, maximum tensile strength, as well as maximum deformation at break. Among all plastomers, the RLLDPE-SBS hybrid combination was observed with the greatest mechanical properties. The three-component blend (B9M2) was found to display the highest tensile strength of 5.42 MPa. All three mixes within the B9 blend show an improved tensile strength compared to plastomer–CR blends, but lower than the plastomer–SBS blends.
- The FTIR results indicate that the chemical composition of the polymeric blends depends greatly on the content of the individual polymers. Increasing the content of one polymer will dominantly produce a higher wavenumber peak for the particular polymer blend.
- Based on the mechanical performance and FTIR results, nine polymeric blends were selected as the optimum polymer content and combinations to further develop hybrid polymer-modified bitumen.
- Rheological tests were conducted on modified bitumen samples to evaluate the effectiveness of the optimum polymeric blends for road applications. The hybrid blend (B9M2) resulted in a significant improvement of elasticity at high temperature (lowest phase angle at 70 °C and greatest G*) among the nine hybrid blends. This is very beneficial in improving the overall pavement performance. The data also shows that the hybrid B9M2 blend enhances the viscoelastic behaviour at both low and high temperature compared to other PMB blends only including two polymers.
- The results for Young’s modulus, maximum tensile strength and elongation, chemical compositions, phase angle, and viscoelastic behaviour from the master curve were highly influenced by the polymer type, content, and combinations, as well as the source of bitumen itself. To fully explore the potential of these hybrid PMBs, it is suggested that further testing is conducted to evaluate storage stability and its enhancement with compatibilizers.

## Figures and Tables

**Table 1:** Test sample combination.

Blend Name	Percentage of Polymers (%) (by Weight of Bitumen)						
	SBS	LDPE	LLDPE	RLLDPE	EVA	SEBS	CR
B1M1	2	3	-	-	-	-	-
B1M2	3.5	2					
B1M3	5	1					
B2M1	2	-	3	-	-	-	-
B2M2	3.5		2				
B2M3	5		1				
B3M1	2	-	-	3	-	-	-
B3M2	3.5			2			
B3M3	5			1			
B4M1	2	-	-	-	3	-	-
B4M2	3.5				2		
B4M3	5				1		
B5M1	2	-	-	-	-	-	20
B5M2	3.5						12.5
B5M3	5						5
B6M1	-	-	-	-	-	2	20
B6M2						3.5	12.5
B6M3						5	5
B7M1	-	-	3	-	-	-	5
B7M2			2				12.5
B7M3			1				20
B8M1	-	-	-	3	-	-	5
B8M2				2			12.5
B8M3				1			20
B9M1	5	-	-	-	3	-	5
B9M2	5				2		5
B9M3	5				1		5

**Table 2:** Physical and chemical properties of materials.

Material	Physical State	Colour	Relative Density (g/cm^3^) (25 °C)	Melting Point (°C)	Source
Styrene Butadiene Styrene (SBS) (B:S ratio of 67/33 linear)	Pellet	White	0.93	N/A	En Chuan Chemical Industries Co. Ltd., China
Styrene Ethylene Butadiene Styrene (SEBS)	Powder	White	0.91	N/A	Sigma-Aldrich, Australia
Ethylene Vinyl Acetate (EVA) (12% vinyl acetate)	Pellet	White	0.933	95	Sigma-Aldrich, Australia
Low-Density Polyethylene (LDPE)	Pellet	White	0.917–0.930	105–115	Sigma-Aldrich, Australia
Linear Low-Density Polyethylene (LLDPE)	Pellet	Light grey	0.918	100–125	Sigma-Aldrich, Australia
Recycled Linear Low-Density Polyethylene (RLLDPE)	Powder	Black	0.91–0.94	110–130	Locally recycled road pits, Australia
Crumb Rubber (CR)	Powder mesh 30	Black	1.15	N/A	Truck tyres only, Local supplier, Australia

**Table 3:** Effect of thermoplastic elastomer addition to CR.

Blend Type	Young’s Modulus		Max. Tensile Strength		Max. Displacement	
	MPa	Increase Factor	MPa	Increase Factor	mm	Increase Factor
CR	1.73	-	0.79	-	19.59	-
B5M1	3.95	2.28	4.52	5.72	62.37	3.18
B5M2	5.07	2.93	4.53	5.73	69.23	3.53
B5M3	10.59	6.12	5.25	6.65	111.55	5.69
B6M1	4.31	2.49	2.60	3.29	46.41	2.37
B6M2	5.47	3.16	3.13	3.96	58.50	2.99
B6M3	10.73	6.20	5.73	7.25	98.57	5.03

**Table 4:** Effect of plastomer addition to CR.

Blend Type	Young’s Modulus		Max. Tensile Strength		Max. Displacement	
	MPa	Increase Factor	MPa	Increase Factor	mm	Increase Factor
CR	1.73	-	0.79	-	19.59	-
B7M3	5.05	2.91	3.30	4.18	39.84	2.03
B7M2	11.02	6.37	3.13	3.96	29.47	1.50
B7M1	63.53	36.72	4.55	5.76	13.01	0.66
B8M3	4.49	2.60	2.69	3.41	39.54	2.01
B8M2	12.34	7.13	3.58	4.53	26.21	1.34
B8M1	70.76	40.90	5.50	6.96	8.23	0.42
